# Supplementary material for: Mutations in PCYT2 disrupt etherlipid biosynthesis and cause a complex hereditary spastic paraplegia
Source: Brain. 2019 Oct 22;142(11):3382–97. doi: 10.1093/brain/awz291 (PMC6821184; doi:10.1093/brain/awz291)
Supplement: awz291_Supplementary_Data [file awz291_supplementary_data.zip › awz291-Suppl_data/Supplementary_Data4.zip!/Supplementary information 4 - plasma lipidomics.zip!/Plasma lipidomics/Control vs Patient2/Statistical plots and data/PE[O] species/Volcano plot_alternative.pdf]

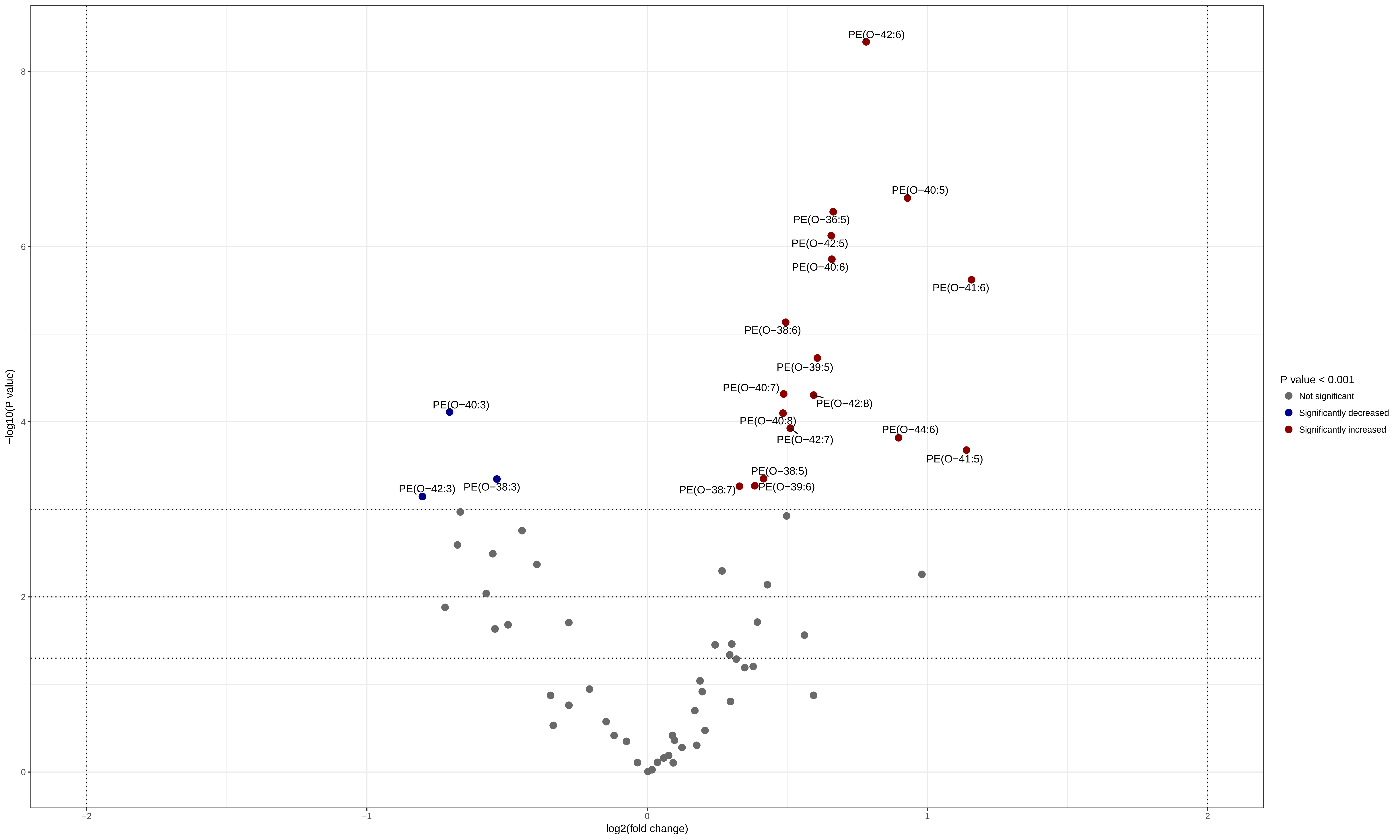

PE(O−42:6)
PE(O−40:5)
PE(O−36:5)
PE(O−42:5)
PE(O−40:6)
PE(O−41:6)
PE(O−38:6)
PE(O−39:5)
PE(O−40:7)
PE(O−42:8)
PE(O−40:3)
PE(O−40:8)
PE(O−42:7)
PE(O−44:6)
PE(O−41:5)
PE(O−38:5)
PE(O−42:3)
PE(O−38:3)
PE(O−38:7)
PE(O−39:6)
8
6
4
2
0
−2
−1
0
1
2
−log10(P value)
log2(fold change)
P value < 0.001
Not significant
Significantly decreased
Significantly increased
